# Defects in cellular sorting and retroviral assembly induced by GGA overexpression

**DOI:** 10.1186/1471-2121-10-72

**Published:** 2009-09-29

**Authors:** Anjali Joshi, Kunio Nagashima, Eric O Freed

**Affiliations:** 1 Virus-Cell Interaction Section, HIV Drug Resistance Program, National Cancer Institute at Frederick, Maryland, USA; 2 Image Analysis Laboratory, Advanced Technology Program, SAIC-Frederick, National Cancer Institute at Frederick, Frederick, Maryland, USA; 3 Department of Biomedical Sciences, Center of Excellence for Infectious Diseases, Paul L. Foster School of Medicine, Texas Tech University Health Sciences Center, El Paso, TX 79905, USA

## Abstract

**Background:**

We previously demonstrated that overexpression of Golgi-localized, γ-ear containing, Arf-binding (GGA) proteins inhibits retrovirus assembly and release by disrupting the function of endogenous ADP ribosylation factors (Arfs). GGA overexpression led to the formation of large, swollen vacuolar compartments, which in the case of GGA1 sequestered HIV-1 Gag.

**Results:**

In the current study, we extend our previous findings to characterize in depth the GGA-induced compartments and the determinants for retroviral Gag sequestration in these structures. We find that GGA-induced structures are derived from the Golgi and contain aggresome markers. GGA overexpression leads to defects in trafficking of transferrin receptor and recycling of cation-dependent mannose 6-phosphate receptor. Additionally, we find that compartments induced by GGA overexpression sequester Tsg101, poly-ubiquitin, and, in the case of GGA3, Hrs. Interestingly, brefeldin A treatment, which leads to the dissociation of endogenous GGAs from membranes, does not dissociate the GGA-induced compartments. GGA mutants that are defective in Arf binding and hence association with membranes also induce the formation of GGA-induced structures. Overexpression of ubiquitin reverses the formation of GGA-induced structures and partially rescues HIV-1 particle production. We found that in addition to HIV-1 Gag, equine infectious anemia virus Gag is also sequestered in GGA1-induced structures. The determinants in Gag responsible for sequestration map to the matrix domain, and recruitment to these structures is dependent on Gag membrane binding.

**Conclusion:**

These data provide insights into the composition of structures induced by GGA overexpression and their ability to disrupt endosomal sorting and retroviral particle production.

## Background

The Gag polyprotein precursors are the key structural elements driving retroviral particle production. The N-terminal matrix (MA) domain of the Gag precursor is important for plasma membrane (PM) targeting and membrane binding. Following Gag targeting, the process of assembly proceeds via Gag multimerization mediated primarily by sequences within the capsid (CA) and nucleocapsid (NC) domains. Finally, the "late" domains in Gag mediate the terminal step in particle production - the pinching off of the virion from the infected cell membrane [1-5]. Concomitant with virus release the particle undergoes maturation, a structural reorganization of the virion that results from a highly concerted catalytic cascade mediated by the viral protease (PR) [5,6].

While the Gag precursor proteins are the sole viral determinants required for the production of immature virus-like particles (VLPs), a number of host factors have been implicated in various steps of the virus assembly and release pathway. Retroviral late domains are known to interact with components of the endosomal sorting machinery. For example, the HIV-1 Gag precursor protein, Pr55^Gag^, contains in its p6 domain a Pro-Thr/Ser-Ala-Pro [P(T/S)AP] motif that binds Tsg101, a component of the endosomal sorting complex required for transport-I (ESCRT-I) [7-10], and a Tyr-Pro-X_n_-Leu (YPX_n_L, where X is any amino acid and n = 1-3 residues) motif that interacts with the ESCRT-associated factor Alix [11-13]. The physiological role of the ESCRT machinery is to promote the biogenesis of vesicles that bud into the lumen of late endosomes to form multivesicular bodies (MVBs) [14]. The recruitment of Tsg101 from the cytoplasm to endosomal membranes occurs via interaction between Tsg101 and hepatocyte growth factor-regulated tyrosine kinase substrate (Hrs) [15-17]. The delivery of cargo proteins to MVBs usually requires the recognition by ESCRT machinery of monoubiquitin moieties attached to the cytoplasmic domains of the cargo. In yeast, disruption of any of the components of the ESCRT complexes and associated factors (known as class E VPS proteins) results in failure of proper sorting of ubiquitinated proteins and induction of an aberrant class E compartment [18-20]. Retroviral Gag proteins are also ubiquitinated [21-23], and while ubiquitination of Gag does not appear to play an essential role in virus budding [24], in the absence of a functional late domain ubiquitin can serve to promote virus release [25].

In addition to serving a well-established role in retrovirus budding, host cell factors have also been reported to function in promoting Gag trafficking to the PM. We previously reported that the phospholipid phosphatidylinositol-(4,5)-bisphosphate [PI(4,5)P_2_] is a key cellular cofactor for HIV-1 Gag targeting to the PM [26] via a direct MA-PI(4,5)P_2 _interaction [27,28]. The clathrin adaptor protein complexes 1, 2, and 3 (AP-1, 2, and 3) [29-31], suppressor of cytokine signaling 1 (SOCS1) [32], the kinesin KIF4 [33,34], staufen 1 [35], and plenty of SH3s (POSH) [36] have all been implicated in Gag targeting to the PM. We demonstrated previously that overexpression of the Golgi-localized, γ-ear containing, Arf-binding (GGA) proteins inhibits the production of HIV-1 and equine infectious anemia virus (EIAV) particles by impairing the association of Gag with membrane [37]. The impairment in Gag-membrane binding induced by GGA overexpression was linked to functional disruption of the endogenous ADP ribosylation factors (Arfs) [37].

The GGA proteins are a family of monomeric clathrin adaptors primarily localized at the trans-Golgi network (TGN), although also reported to be present in late endosomes [38-41]. Three GGA proteins (GGA1, 2, and 3) are expressed in mammals and two in yeast and there is ample evidence demonstrating that these proteins help package cellular cargo into clathrin-coated vesicles [39]. GGAs are comprised of four distinct domains: 1) an N-terminal Vps27, Hrs, and STAM homology (VHS) domain that binds cargo proteins and cargo protein receptors [e.g., cation-dependent mannose 6-phosphate receptors (CD-MPR)] bearing Asp-X-X-Leu-Leu [DXXLL] motifs; 2) a GGA and Tom (GAT) domain that binds Arfs, ubiquitin, and Tsg101; 3) a hinge region that recruits clathrin; and 4) a C-terminal γ-adaptin ear homology (GAE) domain that binds several proteins including rabaptin 5, epsinR, and γ-synergin. The GAT domain is responsible for recruitment of GGA proteins to membrane via interaction with GTP-bound Arf [39].

Previously, we reported that GGA overexpression induces the formation of large swollen vacuolar compartments that sequester Arf proteins. The compartments induced by GGA1, but not those induced by GGA2 or GGA3, also sequester HIV-1 Gag [37]. We and others have also demonstrated in previous studies that overexpression of dominant-negative or full-length components of the ESCRT complexes and associated machinery gives rise to the formation of aberrant compartments that disrupt retroviral particle production. For example, overexpression of full-length Tsg101 (TSG-F) induces a class E-type compartment that potently disrupts HIV-1 budding but has minimal effect on EIAV release [42,43]. Overexpression of the C-terminal portion of Tsg101 (TSG-3') generates aggresome-like structures and severely inhibits the release of HIV-1, murine leukemia virus (MLV), and Rous sarcoma virus (RSV) but does not disrupt EIAV release [42-44]. A dominant-negative, ATPase-deficient mutant of Vps4 is highly disruptive to the release of a number of retroviruses including HIV-1, EIAV, RSV, and feline immunodeficiency virus (FIV) [8,11,25,43,45-47]. It is noteworthy that each of the compartments described above is morphologically distinct and imposes selective defects on specific steps in the retroviral assembly and release pathway. For example, GGA overexpression leads to a defect in Gag trafficking to the PM, whereas TSG-F, TSG-3', and dominant-negative Vps4 overexpression inhibits particle budding and release.

In the current study, we characterized the compartments induced by GGA overexpression with regard to their composition as well as their impact on retrovirus assembly and cellular endosomal sorting pathways. Our data demonstrate that GGA overexpression causes various sorting defects as measured by recycling of CD-MPR, internalization of transferrin receptor (TfR), and the subcellular localization of proteins like Tsg101, ubiquitin, and Hrs. The determinants for HIV-1 Gag sequestration in GGA1-induced compartments were mapped to the MA domain. These data provide novel insights into the defects in cellular sorting and retrovirus assembly induced by GGA overexpression.

## Results

### GGA overexpression induces the generation of enlarged vacuolar compartments that bear Golgi markers

**Figure 1:**
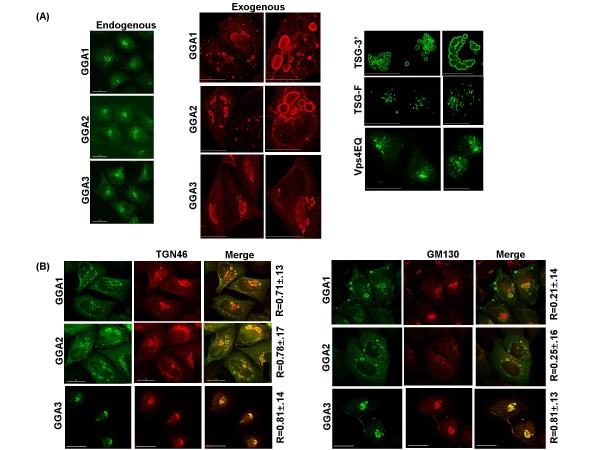
**GGA overexpression leads to accumulation of swollen vacuolar structures that stain positive for Golgi markers**. **(A) **HeLa cells were left untransfected or were transfected with vectors expressing Myc-tagged GGA1, GGA2 or GGA3, HA-tagged TSG-F or TSG-3', or GFP-Vps4EQ. For detection of endogenous GGA proteins (left panels), cells were stained using antibodies specific for GGA1, GGA2 or GGA3. For exogenous GGA or Tsg101 protein detection, cells were stained using anti-Myc or anti-HA antibodies, respectively. Cells were analyzed using the DeltaVision RT microscope. Images shown were obtained from one of 7-8 independent experiments. **(B) **HeLa cells were transfected with the GGA expression vectors, fixed 24 h posttransfection, and stained with antibodies recognizing TGN46 or GM130. Images were acquired using a DeltaVision RT microscope and colocalization was determined using the SoftWoRx colocalization module. Numbers represent average colocalization (R values) ± SD from 10-15 cells. Size bar represents 15 μm.

Previously we showed that GGA overexpression leads to the accumulation of large, swollen vacuolar structures and severely inhibits retrovirus particle production [37]. In the current study, we sought to characterize in detail the phenotype and composition of these GGA-induced structures. As reported previously [48,49], endogenous GGAs predominantly localize to a perinuclear region, with some small puncta also evident at the PM, particularly for GGA3 (Figure 1A, endogenous). All three endogenous GGAs colocalized with Golgi markers (data not shown). In contrast, HeLa cells overexpressing exogenous GGAs showed the accumulation of large, vacuolar compartments (Figure 1A, exogenous). Similar structures were also evident upon overexpression of GFP-tagged GGAs but these tended to be smaller in size (data not shown). These GGA-induced compartments were seen in approximately half of the transfected cells (Figure 1A), with those expressing lower levels of GGA proteins showing a localization pattern similar to that of the endogenous GGAs (data not shown). In parallel, we compared the localization of other cellular proteins which, when overexpressed, also disrupt retroviral particle production and induce the formation of abnormal intracellular structures [19,42,44]. As reported previously, overexpression of full-length Tsg101 (TSG-F) [42] or a GFP-fused, dominant-negative E228Q Vps4 mutant (Vps4EQ) [19,50] induced the formation of swollen endosomal structures known as class E compartments (Figure 1A). In contrast, overexpression of the C-terminal portion of Tsg101 (TSG-3') led to the generation of larger, aggresome-like structures (Figure 1A) [42,44]. To determine whether overexpressed GGA proteins colocalized with each other, we cotransfected cells with vectors expressing the following combinations of tagged GGA proteins: GFP-GGA1 + Myc-GGA2, GFP-GGA1 + Myc-GGA3, and Myc-GGA2 + GFP-GGA3. Microscopic analysis indicated a high degree of colocalization (> 70%) of these combinations of GGA proteins (data not shown).

We next sought to define the composition and subcellular origins of the GGA-induced structures. GGA-overexpressing cells were stained with antibodies specific for the endoplasmic reticulum (ER)-resident proteins calnexin and calreticulin, the trans-Golgi network (TGN) marker TGN46, the Golgi matrix marker GM130, the early endosome marker early endosomal antigen 1 (EEA1), and the late endosome marker CD63. We observed that the GGA-induced structures did not contain ER or late endosome markers (data not shown) but did display a high degree of colocalization with TGN46 (Pearson coefficient of correlation R ~ 0.7-0.8) (Figure 1B). Interestingly, only the GGA3-induced structures stained for GM130 (R ~ 0.8), a detergent-insoluble component of the Golgi matrix peripherally associated with the *cis *compartment (Figure 1B) [51]. These results suggest that GGA overexpression induces the formation of aberrant compartments derived from the Golgi but the specific composition of compartments induced by overexpression of GGA1, GGA2, or GGA3 is not identical.

### Brefeldin A dissociates endogenous GGAs from membranes but does not lead to a disintegration of GGA-induced compartments

**Figure 2:**
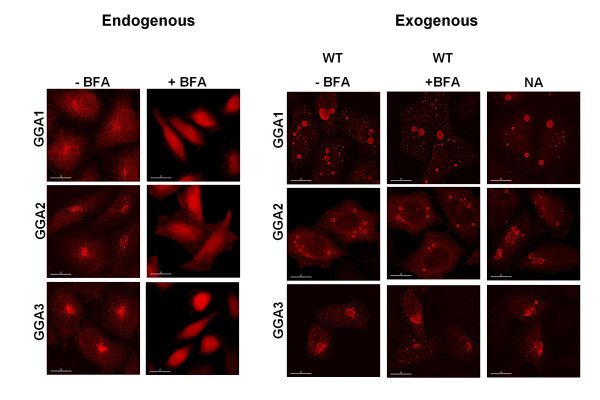
**Treatment with BFA dissociates endogenous GGAs from membranes but does not disrupt the structures induced by GGA overexpression**. HeLa cells were left untransfected, or were transfected with 0.5 μg vectors expressing WT GGAs or NA mutants defective in Arf binding (NA). Approximately 24 h posttransfection, cells were left untreated (-BFA) or were treated with BFA at 4 μg/ml for 1 h (+BFA). Cells were then fixed, stained with anti-GGA antibodies for visualization of endogenous GGAs or anti-Myc antibody for detection of exogenous GGAs, and analyzed by fluorescence microscopy. Size bar represents 15 μm.

Treatment of cells with the fungal toxin brefeldin A (BFA) leads to the dissociation of Arf-dependent coats, including the GGA proteins, from membranes [48,52,53]. It was therefore of interest to determine whether BFA treatment dissociates GGA-induced structures. Untransfected HeLa or GGA-overexpressing cells were treated with BFA at 4 μg/ml for 1 h, fixed and analyzed by fluorescence microscopy. BFA treatment led to the apparent dissociation of endogenous GGAs from membranes, as indicated by a shift in GGA localization from small puncta to a hazy, diffuse pattern (Figure 2, endogenous). In contrast, BFA treatment had no effect on the morphology of GGA-induced structures, and the size and number of swollen compartments remained unchanged (Figure 2, exogenous). Similar results were seen when cells were treated with higher BFA concentrations or for longer periods of time (data not shown). Because the interaction of GGAs with Arf proteins is important for GGA-membrane binding, [39,54], as a control we examined the phenotype of N194A GGA mutants defective in Arf binding. Interestingly, the GGA-N194A mutants also showed massive accumulation of enlarged, swollen, vacuolar compartments (Figure 2, NA panels). Notably, in cells expressing low levels of GGA-N194A protein, a diffuse staining pattern was observed (data not shown), consistent with a previous report [54]. These findings demonstrate that while endogenous GGA proteins dissociate from membranes upon BFA treatment, the morphology of GGA-induced compartments is not altered by BFA treatment. While it is possible that the BFA insensitivity of the GGA-induced compartments is due to a high level of exogenous GGA expression, the BFA data combined with the observation that Arf-binding-deficient GGA mutants still induce these compartments suggest that the association of exogenously expressed GGAs with membrane is not required for the formation and maintenance of GGA-induced structures.

### GGA-induced compartments are positive for the aggresome marker GFP-250

**Figure 3:**
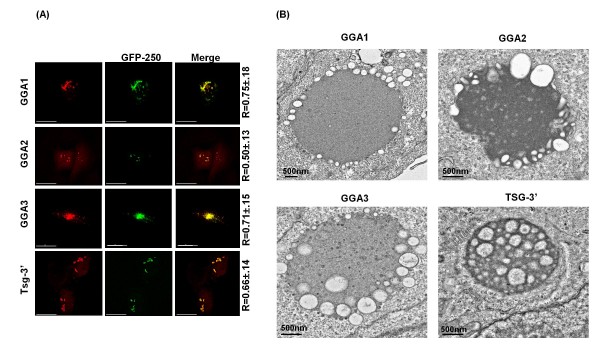
**GGA-induced compartments colocalize with the aggresome marker GFP-250**. **(A) **HeLa cells were cotransfected with vectors expressing Myc-tagged GGAs or HA-tagged TSG-3' and GFP-250 at a 1:1 input DNA ratio. Cells were fixed approximately 24 h posttransfection, stained with anti-Myc or anti-HA antibodies, and analyzed by fluorescence microscopy. Numbers represent mean colocalization (R value) ± SD from 10 cells. Size bar represents 15 μm. **(B) **HeLa cells transfected with GGA or TSG-3' expression vectors were fixed 24 h posttransfection and analyzed by transmission EM. Size bar represents 500 nm.

We next characterized the GGA-induced compartments by determining whether they are aggresome-like structures. Aggresomes are formed in response to protein misfolding or high protein concentrations in the cytoplasm [55]. Other factors such as a change in pH, temperature, ionic strength, or inhibition of a specific degradative pathway can also lead to protein aggregation [56]. GFP-250 is a protein previously characterized to form aggresome-like structures; it is composed of GFP fused to a 250-amino acid fragment of the cytosolic protein and membrane transport anchor, p115 [57]. HeLa cells cotransfected with vectors expressing GFP-250 and Myc-tagged GGA proteins were stained and analyzed by microscopy (Figure 3A). GGA-overexpressing compartments colocalized significantly with GFP-250. As a positive control, we compared the GGA-induced structures with those formed upon TSG-3' expression, as these have been reported to be positive for GFP-250 [44]. Furthermore, EM analysis of GGA- or TSG-3'-overexpressing cells showed an abundance of irregular, electron-dense accumulations of varying densities, shapes and sizes (Figure 3B), which are typical of aggresome-like structures.

### GGA overexpression sequesters cell sorting machinery

**Figure 4:**
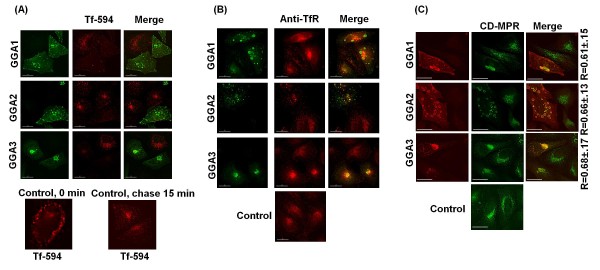
**GGA overexpression perturbs cellular sorting pathways**. **(A) **HeLa cells were transfected with Myc-tagged GGA expression vectors. 24 h posttransfection, cells were washed and incubated for 30 min at 4°C in SFM containing 10 μg/ml Tf-594 then incubated at 37°C for 15 min. Cells were fixed and stained with anti-Myc antibody for transferrin binding and uptake assay. Lower panel: control untransfected cells were incubated with Tf-594 as indicated above and fixed immediately (0 min) or after a 15 min chase. **(B) **HeLa cells transfected with GGA expression constructs were stained with anti-TfR antibody followed by fluorescence microscopy. Lower panel, untransfected cell control. **(C) **HeLa cells were transfected with Myc-tagged GGA expression constructs, stained with anti-CD-MPR antibody followed by microscopy. Numbers represent average colocalization (R value) ± SD from 10 cells. Size bar represents 15 μm.

As shown above, GGA overexpression leads to the accumulation of large swollen compartments. We hence investigated the physiological consequences of GGA overexpression by monitoring epidermal growth factor (EGF) receptor internalization, TfR endocytosis, and CD-MPR recycling. GGA-overexpressing cells were treated with Texas Red-tagged EGF or fluorescently tagged transferrin (Tf-594) conjugates in serum free medium, followed by fluorescence microscopy. GGA overexpression did not significantly alter EGF receptor internalization (data not shown). However, GGA overexpression led to abnormal uptake of transferrin conjugates from the PM. As shown in Figure 4A, in control cells, Tf-594 is rapidly internalized from the PM and can be readily detected in the perinuclear region within 15 minutes. In contrast, cells overexpressing the GGA proteins showed markedly diminished staining with Tf-594 (Figure 4A) at time zero due to sequestration of TfR in GGA-induced compartments as detected by staining of GGA-overexpressing cells with anti-TfR antibody (Figure 4B). GGA overexpression also led to the steady-state accumulation of CD-MPR within GGA-induced structures and its depletion from the periphery (Figure 4C). These data demonstrate that overexpression of GGA proteins disturbs cellular sorting pathways, specifically those involved in TfR but not EGF receptor turnover.

### GGA overexpression alters the localization of cellular endosomal sorting factors important for retrovirus budding

**Figure 5:**
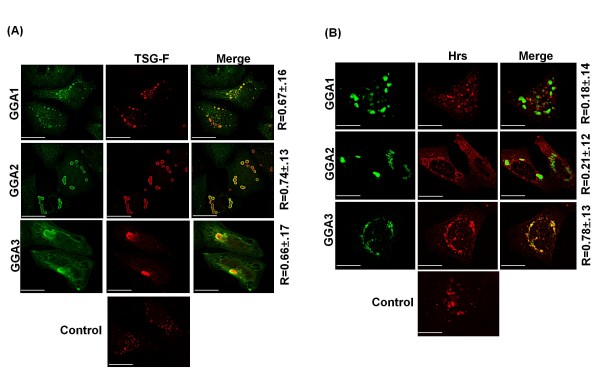
**GGA overexpression alters the localization of endosomal sorting factors**. HeLa cells were transfected with vectors expressing **(A) **Myc-tagged GGAs and HA-tagged TSG-F or **(B) **GFP-tagged GGAs and Myc-tagged Hrs. Cells were fixed approximately 24 h posttransfection, stained with anti-HA and anti-Myc antibodies (A) or anti-Myc antibody (B) followed by microscopy. Numbers represent mean colocalization (R value) ± SD from 8-10 cells. Size bar represents 15 μm.

We next studied the effect of GGA overexpression on the localization of host factors described to be important for retrovirus release. To this end, cells were transfected with vectors expressing HA-tagged Tsg101 and Myc-tagged GGA1, 2, or 3 and analyzed by fluorescence microscopy. Exogenously expressed, full-length Tsg101 (TSG-F) localized to enlarged endosomal structures throughout the cell (Figure 5A, control panel). Interestingly, however, GGA overexpression led to sequestration of exogenous Tsg101 on GGA-induced structures with high degree of colocalization (R ~ 0.7). We next studied the effect of GGA overexpression on Hrs, which recognizes ubiquitinated cargo on early endosomes as part of a complex with signal transducing adaptor molecule 1 (STAM1) and STAM2 [14]. HeLa cells were cotransfected with vectors expressing Myc-tagged Hrs and GFP-tagged GGA proteins (which also induce vacuolar-like structures) and were analyzed by fluorescence microscopy. As expected [58], when expressed alone, Myc-Hrs localized to small puncta typical of early endosomes (Figure 5B, control panel). However, a different localization pattern was observed upon overexpression of the GGA proteins. GGA1 overexpression did not significantly alter the localization pattern of Hrs nor was there appreciable GGA1/Hrs colocalization. In contrast, Hrs appeared to localize to the limiting membrane of GGA2-induced structures and colocalized with GGA3 to a significant extent (R ~ 0.8) (Figure 5B).

### GGA-induced structures sequester ubiquitinated cargo

**Figure 6:**
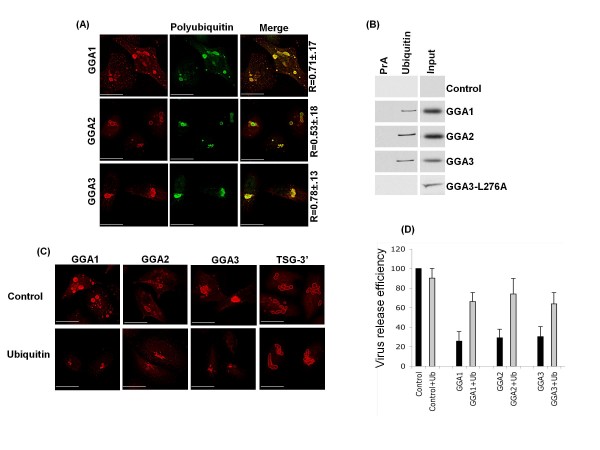
**GGA-induced structures sequester ubiquitinated proteins**. **(A) **HeLa cells were transfected with Myc-tagged GGA expression vectors, fixed, and stained using antibodies specific for the Myc epitope tag or for polyubiquitinated proteins. Numbers represent average colocalization (R values) ± SD from 10 cells. **(B) **GGAs bind directly to ubiquitin. HeLa cells were transfected with vectors expressing the indicated Myc-GGAs. Cell lysates were immunoprecipitated with ubiquitin-agarose or protein A (PrA) agarose beads followed by Western blotting using anti-Myc antibody. **(C) **HeLa cells were transfected with GGA or TSG-3' expression constructs alone or were cotransfected with the ubiquitin expression vector pCW7. Cells were fixed, stained, and analyzed for immunofluorescence. **(D) **HeLa cells were transfected with pNL4-3 along with control or GGA1, GGA2 or GGA3 expression vectors in the presence or absence of control DNA or ubiquitin expression vector. Transfected cells were labeled with [^35^S]Met/Cys, cell and virus lysates immunoprecipitated with HIV-Ig followed by resolution on an SDS-PAGE gel. Error bars indicate mean ± SD, n = 3. Size bar represents 15 μm.

To further define the composition of the GGA-induced structures, we next asked whether they sequester ubiquitin or ubiquitinated cargo. HeLa cells transfected with GGA expression vectors were stained with an antibody that recognizes polyubiquitinated proteins but not free ubiquitin [59]. As shown in Figure 6A, GGA-induced structures were strongly positive for ubiquitinated cargo, especially in the case of GGA1 and GGA3. To confirm the previous observation that GGA proteins bind directly to ubiquitin [49,60-64], lysates from cells expressing Myc-tagged GGAs were immunoprecipitated with ubiquitin-agarose beads followed by immunoblotting with anti-Myc antibody. We observed that all the GGA proteins bound to ubiquitin-agarose but not protein A-agarose beads (Figure 6B). The GGA3 L276A mutant, which displays impaired ubiquitin binding [49] but still induces the formation of aberrant compartments (data not shown), served as a negative control (Figure 6B). Because GGA-induced structures accumulate ubiquitinated proteins (Figure 6A) and GGA overexpression inhibits HIV-1 release [37], we asked whether expression of exogenous ubiquitin could reverse GGA-mediated inhibition of HIV release. HeLa cells were transfected with a ubiquitin expression vector along with GGA expression constructs and analyzed by microscopy. Surprisingly, expression of ubiquitin led to disappearance of the large GGA-induced structures (Figure 6C). GGA proteins were expressed at comparable levels in the presence or absence of ubiquitin overexpression, as determined by Western blot analysis (data not shown). Similar results were obtained when GGA vector DNA was used in a five-fold excess relative to ubiquitin expression plasmid (data not shown). As a control, we examined whether ubiquitin overexpression disrupted the accumulation of compartments induced by TSG-3'. As shown in Figure 6C, ubiquitin overexpression had no effect on the formation of TSG-3'-induced structures. Finally, we investigated whether exogenous ubiquitin expression affected the virus release defect induced by GGA overexpression. Remarkably, we observed that cotransfection of ubiquitin and GGA expression vectors rescued HIV-1 particle production when compared to transfection of GGA vectors alone (Figure 6D). Together, these data indicate that GGA-induced structures sequester ubiquitinated proteins. Moreover, expression of exogenous ubiquitin along with the GGA proteins diminishes the induction of vacuole-like compartments, which correlates with partial rescue of virus release.

### Gag sequestration in GGA1-induced structures requires the MA domain and Gag binding to membrane

**Figure 7:**
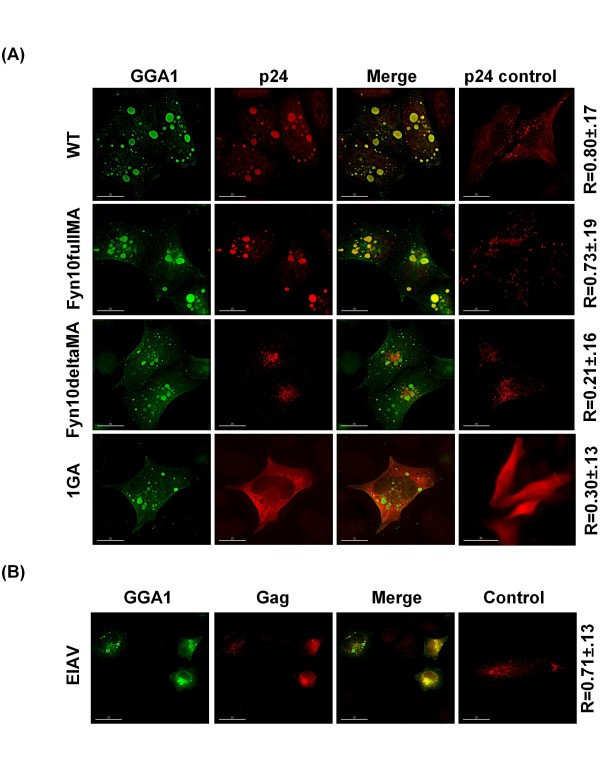
**Sequestration of retroviral Gag proteins in GGA1-induced structures**. **(A) **HeLa cells were transfected with the Myc-tagged GGA1 expression vector along with WT pNL4-3, or the Fyn10fullMA, Fyn10deltaMA, or 1GA derivatives. Cells were fixed approximately 24 h posttransfection, stained using anti-Myc and anti-HIV-1 p24 antibodies followed by immunofluorescence analysis. Numbers represent average colocalization (R value) ± SD from 10 cells. Size bar represents 15 μm. **(B) **HeLa cells were cotransfected with vectors expressing Myc-tagged GGA1 and EIAV Gag. Cells were fixed, stained using anti-Myc and anti-EIAV p26 antibodies and analyzed as indicated above. Numbers represent mean colocalization (R value) ± SD from 7-10 cells.

We demonstrated previously that GGA overexpression inhibits retrovirus particle production, which in the case of GGA1 is in part due to sequestration of HIV-1 Gag in GGA1-induced structures [37]. We also observed that an HIV-1 construct lacking the MA domain (Fyn10deltaMA) is resistant to inhibition mediated by GGA overexpression [37]. We therefore sought to define the determinants in HIV-1 Gag responsible for recruitment into GGA1-induced compartments. HeLa cells were transfected with the GGA1 expression vector along with either full-length pNL4-3; Fyn10fullMA, a pNL4-3 derivative expressing Gag bearing at its N terminus the ten-amino-acid membrane-targeting signal from Fyn [65]; Fyn10deltaMA, a pNL4-3 derivative expressing Gag bearing the ten amino acid residues from Fyn and lacking the MA domain [66]; or pNL4-3/1GA, which expresses a non-myristylated Gag mutant [67]. Cells were then stained using an anti-HIV-1 p24 (CA) antibody and the degree of colocalization between GGA1 and Gag was quantified. Interestingly, GGA1-induced structures sequestered WT and Fyn10fullMA Gag but not Fyn10deltaMA Gag suggesting a requirement for the MA domain in Gag sequestration (Figure 7A). Moreover, the GGA1-induced structures did not colocalize with 1GA Gag, demonstrating that Gag recruitment into GGA1-induced structures required Gag-membrane binding (Figure 7A). Finally, we asked whether non-HIV-1 Gag proteins are also sequestered in GGA1-induced structures. Interestingly, EIAV Gag showed a high degree (R > 0.8) of colocalization with GGA1-induced structures (Figure 7A) whereas MLV Gag demonstrated a lower level of colocalization (R < 0.4) with these structures (data not shown).

## Discussion

Previously we showed that GGA overexpression modulates retrovirus assembly by inhibiting Gag targeting to the PM. GGA overexpression also induced the accumulation of large vacuolar structures, which in the case of GGA1 sequestered HIV-1 Gag [37]. In the current study, we investigated in detail the morphology and composition of GGA-induced compartments and the impact of their formation on normal cellular physiology and retrovirus release. We observed that GGA-induced compartments stained positively for Golgi and aggresome markers, and GGA overexpression caused defects in endosomal sorting. Moreover, GGA overexpression also altered the localization of various host proteins important for retrovirus assembly. Overexpression of ubiquitin led to a dissolution of the GGA-induced structures and a partial rescue of HIV-1 release. Study of determinants for HIV-1 Gag sequestration in GGA1-induced compartments demonstrated a role for the MA domain. Thus, GGA overexpression not only inhibits retrovirus assembly and Gag localization but also alters the cellular sorting pathways important for normal cellular physiology.

GGA proteins when expressed exogenously in cells whether transiently or stably have been reported to show a localization pattern similar to that of their endogenous counterparts [48,49]. Indeed, tagged GGA expression vectors have been widely used in the field of cell biology to substitute for the use of GGA antibodies. However, we observe defects in endosomal sorting induced by even moderate levels of GGA overexpression and the formation of vacuolar compartments in which the exogenously expressed GGA proteins are localized. This phenotype is similar to the GGA expression pattern reported previously, namely, "compaction and fizzling" of Golgi stacks and accumulation of fragmented vacuolar-like blobs that contain Golgi markers [52,68,69]. GGA overexpression also led to striking changes in TfR distribution and recycling of CD-MPR. Following ligand binding to the TfR, both receptor and ligand are rapidly internalized and transported to acidic compartments in which the receptor is released and recycled back to the cell surface [70,71]. However, in cells overexpressing the GGA proteins, there was no evidence of the bright cell surface staining with fluorescently tagged transferrin that is observed in cells not overexpressing the GGAs. This loss in transferrin binding was due to sequestration of TfR in GGA-induced compartments. GGA overexpression led to the accumulation of CD-MPR in the Golgi with a concomitant depletion from the periphery. This alteration in CD-MPR recycling has also been described previously in cells expressing a dominant-negative fragment of GGA1 comprising the VHS-GAT domain but lacking the hinge and GAE domains [72].

GGA overexpression also led to alterations in the subcellular localization of several host proteins implicated in retrovirus assembly and release. We previously observed that GGA overexpression led to sequestration of Arfs [37]. Moreover, GGA overexpression also caused a shift in the localization of overexpressed Tsg101 and Hrs (Figure 5). Of particular interest were the observations that GGA-induced compartments sequestered ubiquitinated cargo and providing exogenous ubiquitin not only resolved the GGA-induced structures but also rescued HIV-1 release (Figure 6). We speculate that GGA-induced structures are formed as a result of misfolded protein accumulation, which due to ubiquitin sequestration are incapable of being targeted for proteasomal degradation [73,74]. Providing exogenous ubiquitin could facilitate destruction of misfolded proteins leading to the disappearance of GGA-induced structures. Interestingly, this phenomenon was not observed for TSG-3'-induced structures despite their ability to sequester ubiquitinated cargo. The observations that BFA did not disrupt the GGA-induced compartments and that these compartments were still induced by the Arf-binding-deficient GGA mutants (Figure 2) argue that formation of the GGA-induced structures is independent of Arf binding and GGA membrane association. These GGA-induced structures possess some properties of aggresomes [for example, they stain with the aggresome marker GFP-250 (Figure 3)] but are clearly distinct in terms of composition and inhibitory activity from the aggresome-like structures induced by TSG-3'. Most notably, TSG-3'-induced structures primarily block virus budding and do not interfere with EIAV release [42,43], whereas GGA-induced structures impair Gag association with membrane and display inhibitory activity against HIV-1 and EIAV ([37]; this study). The distinct phenotypes of these compartments is likely due to differences in the proteins that they sequester. Purification of these aberrant structures and proteomic characterization of their composition would potentially provide additional insights into host cell machinery required for Gag trafficking to the plasma membrane and virus budding.

Although the three mammalian GGA proteins are closely related, in our studies they have shown some notable differences: 1) depletion of GGA3, and to a lesser extent GGA2, stimulated HIV-1 release whereas GGA1 depletion did not [37]. 2) Whereas the compartments induced by overexpression of each of the three GGA proteins stained positively for the TGN marker TGN46, only those induced by GGA3 were positive for the detergent-insoluble cis-Golgi marker GM130 (Figure 1). 3) Compartments induced by GGA1 trapped HIV-1 Gag, whereas those induced by GGA2 or GGA3 did not [37]. It remains to be determined whether the GGA proteins interact directly with Gag or whether differential association with other host cell factors could explain the ability of GGA1 but not GGA2 or GGA3 overexpression to trap Gag. In this study, we observed that Fyn10deltaMA and the non-myristylated HIV-1 Gag mutant were not sequestered in GGA1-induced compartments, indicating a requirement for the MA domain and Gag membrane binding in Gag trapping.

In contrast to the Gag sequestration data, in virus release assays both Fyn10FullMA (Joshi and Freed, unpublished) and Fyn10deltaMA [37] were resistant to inhibition mediated by GGA overexpression. This observation suggests that Gag sequestration is not the sole cause of inhibition mediated by GGA1 overexpression. It is also possible that the Fyn targeting signal, which confers strong membrane binding ability, is able to rescue the inhibition. Interestingly, EIAV particle production is also inhibited by GGA overexpression [37] and EIAV Gag is sequestered in GGA1-induced structures (Figure 7B). Moreover, while overexpression of GGA2 and GGA3 mutants defective in Arf binding (the GGA-NA mutants) was no longer inhibitory to HIV-1 and EIAV release, the GGA1-NA mutant was still capable of inhibition and Gag sequestration [37]. Overall, these findings indicate intriguing differences in the mechanisms by which overexpression of the three GGA proteins inhibit retroviral particle production.

## Conclusion

In the current study, we extend our previous findings and characterize in detail the structures induced by GGA overexpression with respect to their impact on cellular sorting pathways and their ability to inhibit retroviral particle production. We observe that the compartments induced by GGA overexpression are distinct from those formed by dominant-negative components of the ESCRT pathway, both in terms of their effect on cellular functions and with respect to the step in the retrovirus assembly and release pathway that they disrupt. A better understanding of these host components will not only further our knowledge of cellular sorting processes but also help define the requirements for host factors in retrovirus assembly and release.

## Methods

### Cell culture and transfections

HeLa cells were cultured in DMEM supplemented with 5% fetal bovine serum (FBS) and 2 mM glutamine. All transfections were performed using Lipofactamine2000™ reagent (Invitrogen) as per the manufacturer's instructions.

### Reagents

Plasmids expressing full-length Myc-tagged GGA proteins were kindly provided by J. Bonifacino (NIH, Bethesda MD) [39,48,49]. The GGA3 mutant defective in ubiquitin binding (L276A) [49] was constructed by site-directed mutagenesis. The full-length HIV-1 proviral clone pNL4-3 [75] and the myristylation-deficient mutant pNL4-3/1GA [67] have been previously reported. The ubiquitin expression vector pCW7 [76] was provided by R. Kopito (Stanford University, CA). An HIV-1 derivative bearing at its N terminus ten amino acids from the membrane targeting sequence of Fyn (Fyn10fullMA) [65] and its derivative lacking the MA domain (Fyn10deltaMA) [66] have been described. HA-tagged Tsg101 expression constructs TSG-F and TSG-3' have been reported [42,77]. The Hrs expression vector was constructed by amplifying the Hrs coding region from a cDNA library by PCR and inserting the amplified fragment between the BamHI and XbaI sites of pcDNA3.1. The EIAV Gag expression vector pPRE/Gag has been reported previously [43,78]. The aggresome marker GFP-250 [57] was kindly provided by E. S. Sztul (University of Alabama at Birmingham, Birmingham AL). The pEGFPhVPS4A (E228Q) expressing an ATPase-deficient mutant of VPS4A fused to GFP was a gift from P. Woodman (University of Manchester, UK) [50]. The anti-GGA1 antibody was a kind gift from R. Kahn (Emory University, Atlanta, GA) [52]; anti-GGA2 and GGA3 antibodies were purchased from BD Biosciences. The mouse anti-GM130 antibody was from Transduction Laboratories (Lexington, KY) and sheep anti-TGN46 was from Serotec (Oxford, United Kingdom). EIAV anti-p26 antibody was kindly provided by R. Montelaro (University of Pittsburg). EGF-Texas Red and Transferrin Alexa Fluor 594 (Tf-594) conjugates were from Molecular Probes (Invitrogen). The anti-polyubiquitin antibody was from Biomol (clone Fk1). The anti-CD-MPR antibody (clone 22d4) was obtained from University of Iowa Developmental Studies Hybridoma bank and anti-TfR antibody was from Zymed. BFA was purchased from Calbiochem. Protein A beads were from Invitrogen and ubiquitin-agarose beads were obtained from Sigma.

### Immunofluorescence and EM analysis

Immunostaining of cells was performed as described [79], with minor modifications. Cells seeded onto Nunc Lab-Tek II chamber slides were rinsed with phosphate-buffered saline (PBS) and fixed with 3.7% formaldehyde in 100 mM sodium phosphate buffer (pH 7.2) for 20 min. Following fixation, cells were thoroughly rinsed with PBS, permeabilized using 0.1% Triton X-100/PBS for 2 min, and incubated for 10 min with 0.1 M glycine/PBS to quench the remaining aldehyde residues. Cells were then blocked with 3% BSA/PBS for 30 min, followed by incubation for 1 h with primary antibody appropriately diluted in 3% BSA/PBS. After 3 washes in PBS, cells were incubated for 30 min with secondary antibody diluted in 3% BSA/PBS. Cells were then washed and mounted using Aqua Poly/Mount (Polysciences Inc). Images were acquired with a DeltaVision RT microscope. To quantify colocalization, we calculated the Pearson correlation coefficient (R) values, which are standard measures of colocalization [80]. The R values were calculated using the softWoRx colocalization module which generates a "colocalized" image from two channels. A scatter plot of the two intensities on a pixel-by-pixel basis was plotted and the R value calculated by dividing the covariances of each channel by the product of their standard deviations. For EM analysis, transfected cells were fixed using buffer containing 2% glutaraldehyde and 100 mM sodium cacodylate and stored at 4°C. Samples were then sectioned and analyzed by transmission EM [67].

### EGF and TfR internalization assays

24 h posttransfection, cells were washed three times with serum free medium (SFM) containing 20 mM HEPES and 1% BSA and incubated for 1 h at 37°C in the same medium. Cells were then placed on ice for 5 min followed by incubation for 30 min at 4°C in SFM containing 5 μg/ml EGF-Texas Red or 10 μg/ml Tf-594 (Molecular Probes). Cells were washed three times with PBS and incubated in cell culture medium at 37°C for 15 min for TfR and 30 min for EGF receptor. Finally, cells were washed with PBS, fixed and mounted using DAPI-containing medium for EGF receptor assays.

### Metabolic labeling and immunoprecipitation

The protocol for radiolabeling and immunoprecipitation of cell and virus lysates has been described in detail previously [67]. Briefly, transfected cells were starved for 30 min in labeling media lacking Met and Cys. Thereafter, cells were incubated for 2-3 h in labeling medium supplemented with FBS and [^35^S]Met/Cys. Culture supernatants were ultracentrifuged at 100,000 × g for 45 min, cell and virus lysates were immunoprecipated with HIV-Ig, resolved by SDS-PAGE followed by PhosphorImager analysis. Virus release was calculated as the percentage of virion-associated p24 (CA) relative to total (virion + cell-associated) Gag. Virus release efficiency = virion p24/(cell associated Pr55^Gag ^+ cell-associated p24+virion-associated p24) × 100.

## Abbreviations

Alix: ALG-2 interacting protein X; AP: adaptor proteins; Arfs: ADP ribosylation factors; BFA: brefeldinA; BSA: bovine serum albumin; CA: capsid; CD-MPR; cation-dependent mannose 6-phosphate receptor; DMEM: Dulbecco-modified Eagle's medium; EEA: early endosomal antigen; EGF: epidermal growth factor; EIAV: equine infectious anemia virus; EM: electron microscopy; ER: endoplasmic reticulum; ESCRT: endosomal sorting complex required for transport; FBS: fetal bovine serum; FIV: feline immunodeficiency virus; GAE: γ-adaptin ear homology domain; GAT: GGA and Tom domain; GGA: Golgi-localized, γ-ear containing, Arf-binding; GM130: Golgi matrix marker 130; Hrs: hepatocyte growth factor-regulated tyrosine kinase; MA: matrix; MLV: murine leukemia virus; MVB: multivesicular body; NC: nucleocapsid; PBS: phosphate-buffered saline; PI(4,5)P_2_: phosphatidylinositol-(4,5)-bisphosphate; PM: plasma membrane; POSH: plenty of SH-3s; PR: protease; P(T/S)AP: Pro-Thr/Ser-Ala-Pro; RSV: Rous sarcoma virus; SDS-PAGE: sodium dodecyl sulfate polyacrylamide gel electrophoresis; SFM: serum-free medium; STAM: signal transducing adaptor molecule; TfR: transferrin receptor; TGN: trans-Golgi network; Tsg101: tumor susceptibility gene 101; SOCS1: suppressor of cytokine signaling 1; VHS: Vps27, Hrs and STAM homology; VLP: virus-like particle.

## Authors' contributions

AJ performed the experiments and participated in the design of experiments, the interpretation of the data, and the writing of the manuscript. KN performed EM analysis. EOF participated in the design of experiments, the interpretation of the data, and the writing of the manuscript. All authors read and approved the final version of the manuscript.
